# DDBJ update in 2024: the DDBJ Group Cloud service for sharing pre-publication data

**DOI:** 10.1093/nar/gkae882

**Published:** 2024-10-09

**Authors:** Yuichi Kodama, Takeshi Ara, Asami Fukuda, Toshiaki Tokimatsu, Jun Mashima, Takehide Kosuge, Yasuhiro Tanizawa, Tomoya Tanjo, Osamu Ogasawara, Takatomo Fujisawa, Yasukazu Nakamura, Masanori Arita

**Affiliations:** Bioinformation and DDBJ Center, National Institute of Genetics, 1111 Yata, Mishima, Shizuoka 411-8540, Japan; Bioinformation and DDBJ Center, National Institute of Genetics, 1111 Yata, Mishima, Shizuoka 411-8540, Japan; Bioinformation and DDBJ Center, National Institute of Genetics, 1111 Yata, Mishima, Shizuoka 411-8540, Japan; Bioinformation and DDBJ Center, National Institute of Genetics, 1111 Yata, Mishima, Shizuoka 411-8540, Japan; Bioinformation and DDBJ Center, National Institute of Genetics, 1111 Yata, Mishima, Shizuoka 411-8540, Japan; Bioinformation and DDBJ Center, National Institute of Genetics, 1111 Yata, Mishima, Shizuoka 411-8540, Japan; Bioinformation and DDBJ Center, National Institute of Genetics, 1111 Yata, Mishima, Shizuoka 411-8540, Japan; Bioinformation and DDBJ Center, National Institute of Genetics, 1111 Yata, Mishima, Shizuoka 411-8540, Japan; Bioinformation and DDBJ Center, National Institute of Genetics, 1111 Yata, Mishima, Shizuoka 411-8540, Japan; Bioinformation and DDBJ Center, National Institute of Genetics, 1111 Yata, Mishima, Shizuoka 411-8540, Japan; Bioinformation and DDBJ Center, National Institute of Genetics, 1111 Yata, Mishima, Shizuoka 411-8540, Japan; Bioinformation and DDBJ Center, National Institute of Genetics, 1111 Yata, Mishima, Shizuoka 411-8540, Japan

## Abstract

The Bioinformation and DNA Data Bank of Japan Center (DDBJ Center, https://www.ddbj.nig.ac.jp) provides public databases that cover a wide range of fields in life sciences. As a founding member of the International Nucleotide Sequence Database Collaboration (INSDC), the DDBJ Center accepts and distributes nucleotide sequence data ranging from raw reads to assembled and annotated sequences with the National Center for Biotechnology Information and the European Bioinformatics Institute. Besides INSDC databases, the DDBJ Center provides databases for functional genomics (Genomic Expression Archive), metabolomics (MetaboBank), human genetic variations (TogoVar-repository) and human genetic and phenotypic data (Japanese Genotype-phenotype Archive). These database systems have been built on the National Institute of Genetics supercomputer, which is also a platform for the DDBJ Group Cloud (DGC) services for sharing and analysis of pre-publication data among research groups. This paper reports recent updates on the databases and the services of the DDBJ Center, highlighting the DGC service.

## Graphical Abstract

**Graphical Abstract:**
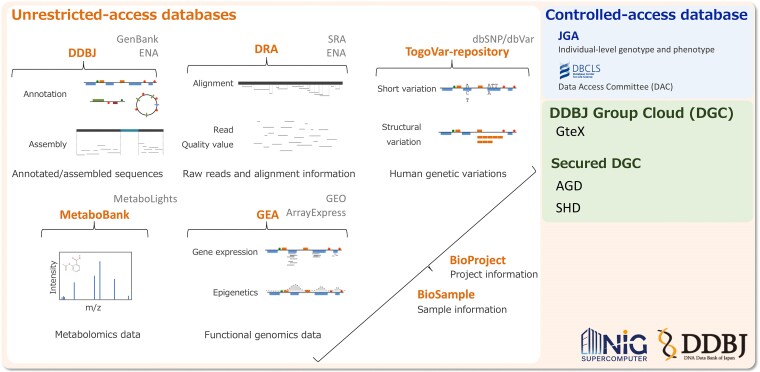

## Introduction

The Bioinformation and DNA Databank of Japan Center (DDBJ Center, https://www.ddbj.nig.ac.jp) of the National Institute of Genetics (NIG) is one of the leading resources of public biological data (1). As a founding member of the International Nucleotide Sequence Database Collaboration (INSDC) (2), the DDBJ Center has been accepting nucleotide sequence data ranging from raw reads to assembled sequences with functional annotation, issuing accession numbers and distributing them in collaboration with the National Center for Biotechnology Information (NCBI) (3) and the European Nucleotide Archive (ENA) at the European Bioinformatics Institute (EBI) (4). In addition to the INSDC databases, the DDBJ Center provides the Genomic Expression Archive (GEA) (5) for quantitative data from functional genomics experiments (e.g. gene expression and epigenetics) and MetaboBank (1) for metabolomics raw data. In September 2024, TogoVar-repository was launched to accept submissions of human genetic variations, issue accession numbers and distribute them. Short variants such as single nucleotide variants (SNVs) and small-scale insertions and deletions (INDELs) are exchanged with the NCBI dbSNP (3), and large structural variations (>50 bp) are exchanged with the NCBI dbVar (6). TogoVar-repository complements TogoVar of the Database Center for Life Science (DBCLS), a service to catalog variants observed in Japanese populations with frequencies and annotations for variant interpretation (7). The BioProject and BioSample records organize related multi-omics datasets across databases for easier access (8). The DDBJ Center supports the scientific community by providing unrestricted access to large-scale biological data in accordance with the FAIR (Findable, Accessible, Interoperable and Reusable) data principle (9). The DDBJ Center is one of the Global Core Biodata Resources, a collection of data resources recognized as critical to life science and biomedical research worldwide (https://globalbiodata.org/).

In addition to these unrestricted-access databases, the DDBJ Center services a controlled-access database, the Japanese Genotype–phenotype Archive (JGA), in collaboration with DBCLS (10). JGA stores genotype and phenotype data from human individuals who have signed consent agreements authorizing data usage for specific research only. The DBCLS Data Access Committee (DAC) reviews data submission and usage requests in accordance with the human data sharing guidelines (https://humandbs.dbcls.jp/en/guidelines). In April 2024, the DAC was transferred from the National Bioscience Database Center (NBDC) of the Japan Science and Technology (JST) to DBCLS.

As vast amounts of data are produced in various fields of life science, sharing and analysis of large and complex datasets with research collaborators become critical to advance research. The DDBJ Group Cloud (DGC) service has been operated for sharing and analysis of pre-publication data among research groups in the NIG supercomputer where our public data resources are maintained. Validation services for the public database submissions improve the quality of data shared in DGC.

In this article, we report updates to the databases and the services of the DDBJ Center, highlighting the DGC service. All resources are available at https://www.ddbj.nig.ac.jp and the data are accessible at ftp://ftp.ddbj.nig.ac.jp and https://ddbj.nig.ac.jp/public/.

## Data contents and services

### Data contents: unrestricted- and controlled-access databases

In 2023, DDBJ accepted 5822 submissions for nucleotide sequences, among which 74.5% were contributions from domestic Japanese research groups. Among annotated bacterial genomes submitted in 2023, 98.5% were annotated by using the prokaryotic genome annotation pipeline DFAST (11). DDBJ has released all public DDBJ/ENA/GenBank nucleotide sequence data periodically. The latest release of June 2024 contains 4 556 178 970 sequences and 31 877 785 876 529 bp, and DDBJ contributed 4.21% of the sequences and 2.22% of the bases. The DDBJ Sequence Read Archive (DRA) accepted 94 299 runs of high-throughput sequencing data in 2023. As of August 2024, DRA provides 19.4 PB of sequencing data in SRA (17.9 PB) and FASTQ (1.5 PB) formats. GEA accepted 79 submissions of functional genomics data in 2023, and 240 experiment datasets are available via the FTP site (ftp://ftp.ddbj.nig.ac.jp/ddbj_database/gea) as of August 2024. MetaboBank accepted 12 studies of metabolomics data in 2023, and 135 studies are available via the FTP site (ftp://ftp.ddbj.nig.ac.jp/metabobank) as of August 2024. MetaboBank provides raw metabolomics data in submitted vendor-specific (e.g. WIFF and RAW) and open-standard (e.g. mzML) formats as well as in a general-purpose format ‘mzB’ developed by the Japanese company Reifycs. A free viewer and Python/C#/Java libraries for viewing and accessing the mzB file contents are available at Reifycs. More details are explained in our web page (https://www.ddbj.nig.ac.jp/metabobank/mzB-e.html) and we welcome feedback from users.

JGA accepted 72 studies amounting to 97 TB of data in 2023, and 409 studies, 804 081 samples and 1 PB of human data are available under controlled access as of August 2024. Summaries of JGA studies and datasets are available without restriction on the DDBJ Search (https://ddbj.nig.ac.jp/search) and the DBCLS website (https://humandbs.dbcls.jp/en/data-use/all-researches). To access personal raw data, users are required to submit data usage requests to the DBCLS DAC. In 2023, there were 106 such requests. An overall statistics is available on our website (https://www.ddbj.nig.ac.jp/statistics/index-e.html).

### TogoVar-repository for variant submission

TogoVar of DBCLS provides genetic variations of >200 000 Japanese individuals with additional information of allele frequencies and clinical significance, based on multiple resources such as gnomAD, ClinVar and dbSNP (7). TogoVar uses non-redundant variant IDs for the purpose of data integration. On the other hand, a repository accepting variant submission needs to assign distinct identifiers (accession numbers) for possibly identical variants if submitters provide them independently. For this purpose, TogoVar-repository accepts human genetic variants and allele/genotype frequencies in two categories and issues accession numbers with distinct prefixes: short variants (≦50 bp) such as SNVs and small-scale INDELs, and large structural variants (>50 bp) such as copy number variations and chromosomal translocations. Both categories are exchanged with dbSNP and dbVar at NCBI (3), respectively. As of September 2024, two new studies are publicly available via our FTP site (ftp://ftp.ddbj.nig.ac.jp/togovar). The first is structural variants detected in one of the 1000 Genomes Japanese individuals (study accession dstd1), and the other is 200 million SNVs/INDELs with allele frequencies obtained from whole genome sequencing data of 9287 Japanese healthy individuals from the Ryukyu Islands and other parts of Japan (study accession dstd2) (12).

### INSDC spatiotemporal metadata standards

INSDC is committed to improve FAIRness of nucleotide sequence records with metadata and has introduced a new standard in March 2023: mandating the isolation time and location of the sample, unless a valid exemption is declared (https://www.insdc.org/news/insdc-spatiotemporal-metadata-minimum-standards-update-03-03-2023/). Another update was the standard for reporting missing values in April 2023 to allow for ‘reporting levels’ so that submitters can describe reasons for missing metadata (https://www.insdc.org/submitting-standards/missing-value-reporting). All records submitted to the BioSample database will include at least the geo-location and year of their collection, or an exemption should be filed. INSDC also introduces the minimum standards to other sequence records over coming years. This change will significantly increase traceability and usability of INSDC data by enriching contextual metadata of samples and their sequence records. For more details, readers are referred to the INSDC article of the same issue.

## DDBJ Group Cloud

**Figure 1.**
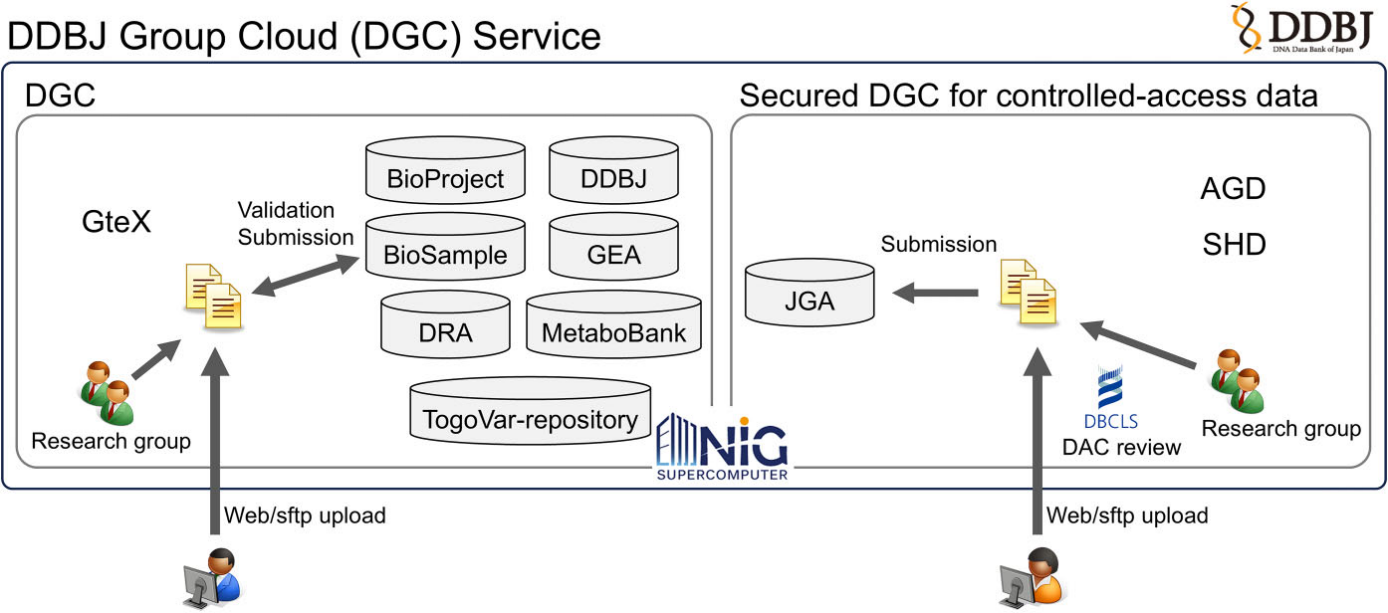
The DGC services for sharing personal raw data [right, two projects the AMED Genome Group Sharing Database (AGD) and the Strategic Innovation Promotion Program Healthcare Group Sharing Database (SHD)] and other data [left, one project Green Technologies for Excellence (GteX)] among research groups in the NIG supercomputer. Users upload data through web/sftp and share the data with specific research groups. The validation and submission services as well as public datasets of the public databases maintained in the NIG supercomputer are available in DGC. In the secured DGC, personal data are uploaded and used after approval from the DBCLS DAC.

As more national funds are invested in science and technology, the government is more concerned about open-and-close strategies of scientific information. Traditionally, research data are stored in institutional computing centers with necessary computational tools, but maintaining similar analysis tools at different centers or transferring large datasets among centers is inefficient. For better sharing and analysis of pre-publication data among research groups, the DGC service has been active since 2017, initially for controlled access data (Figure 1). DGC offers the following advantages over the institutional computing centers.

- Users can control access to their uploaded pre-publication data.
- Public datasets including INSDC and 1000 Genomes are locally accessible.
- Validation services of INSDC (for public data submission) are available.
- Permanent accession numbers are assigned on condition of data publication.
- Bioinformatics tools of the NIG supercomputing system are available.

Since April 2024, a DGC service has been expanded to the sharing of microbial and plant multi-omics datasets generated by the Biomanufacturing Area group of the GteX Program of JST (https://www.jst.go.jp/gtex/en/field/bio.html) (Figure 1). In the GteX version of DGC, users can validate and standardize their data through a graphical user interface. For example, the BioSample validation service validates plant sample metadata by using the ‘plant package’ offering necessary and recommended attributes to describe plant samples, ensuring that the metadata are reusable to research collaborators. Data will be publicly shared only after official DDBJ submission and data release so that any researcher can access data through permanent accession numbers. It should be noted that the assigned accession numbers should not be cited before the publication of full data (see our INSDC data release policy at https://www.insdc.org/submitting-standards/insdc-status-document/).

DGC is not a free service, and customizing and operational costs are borne by the DGC subscribers. In addition, the issuance of DGC accounts is restricted to Japanese domestic researchers or their collaborators, because the NIG supercomputer is operated under the Foreign Exchange and Foreign Trade Act of Japan (https://sc.ddbj.nig.ac.jp/en/application/). This does not necessarily exclude overseas researchers, although they need to establish joint research with Japanese researchers for subscription. Data on DGC can be designated as public access by the users as in other cloud systems.

For personal data requiring controlled access, a secure version of DGC is provided. In the secure DGC, data upload and access are reviewed beforehand by the DBCLS DAC in accordance with the NBDC human data sharing guidelines. The DAC ensures ethical data sharing and alleviates a further review at the time of JGA submission. Two secured DGC services have been in operation: the AMED Genome Group Sharing Database (AGD, 2018–) and the Strategic Innovation Promotion Program Healthcare Group Sharing Database (SHD, 2021–2024) (https://gr-sharingdbs.dbcls.jp/, Japanese language only). In AGD, genome and transcriptome sequencing data, and spatial gene expression data of healthy and disease Japanese individuals, have been shared, and GPU (Graphics Processing Unit)-accelerated mapping and variant calling workflows based on the GATK Best Practices (13) are available. Whole genome sequencing (WGS) data of 9830 healthy Japanese individuals in AGD were already analyzed by joint call with WGS data of 1000 Genomes individuals. The aggregated 200 million variants and their frequencies are now available without access restriction at TogoVar (12).

In SHD, gut microbiome sequencing data and phenotypes of 1267 Japanese individuals have been shared and analyzed by academic and company researchers through a project-specific graphical user interface. All SHD datasets were transferred to JGA upon the project closure in April 2024, and a new project is being planned. We strongly encourage data publication from the public databases upon project closure, to promote data sharing with wider scientific communities and prevent data dissipation or loss. Being a subscription service, however, it is not mandatory for DGC users to make everything public at the end of service subscription.

## Future direction

We will improve the DDBJ Search to cover all public records of the databases at the DDBJ Center to increase findability. The DDBJ Center will collaborate with DBCLS to improve the variation services TogoVar-repository and TogoVar, and the controlled-access database services JGA and the DAC application system.

## Data Availability

All resources are available at https://www.ddbj.nig.ac.jp and the data are accessible at ftp://ftp.ddbj.nig.ac.jp and https://ddbj.nig.ac.jp/public/.
